# Mapping the medical outcomes study HIV health survey (MOS-HIV) to the EuroQoL 5 Dimension (EQ-5D-3 L) utility index

**DOI:** 10.1186/s12955-019-1135-8

**Published:** 2019-05-10

**Authors:** Yuan Shi, Jennifer Thompson, A. Sarah Walker, Nicholas I. Paton, Yin Bun Cheung, for the EARNEST Trial Team, E. Agweng, P. Awio, G. Bakeinyaga, C. Isabirye, U. Kabuga, S. Kasuswa, M. Katuramu, C. Kityo, F. Kiweewa, H. Kyomugisha, E. Lutalo, P. Mugyenyi, D. Mulima, H. Musana, G. Musitwa, V. Musiime, M. Ndigendawan, H. Namata, J. Nkalubo, P. Ocitti Labejja, P. Okello, P. Olal, G. Pimundu, P. Segonga, F. Ssali, Z. Tamale, D. Tumukunde, W. Namala, R. Byaruhanga, J. Kayiwa, J. Tukamushaba, S. Abunyang, D. Eram, O. Denis, R. Lwalanda, L. Mugarura, J. Namusanje, I. Nankya, E. Ndashimye, E. Nabulime, D. Mulima, O. Senfuma, G. Bihabwa, E. Buluma, P. Easterbrook, A. Elbireer, A. Kambugu, D. Kamya, M. Katwere, R. Kiggundu, C. Komujuni, E. Laker, E. Lubwama, I. Mambule, J. Matovu, A. Nakajubi, J. Nakku, R. Nalumenya, L. Namuyimbwa, F. Semitala, B. Wandera, J. Wanyama, H. Mugerwa, A. Lugemwa, E. Ninsiima, T. Ssenkindu, S. Mwebe, L. Atwine, H. William, C. Katemba, S. Abunyang, M. Acaku, P. Ssebutinde, H. Kitizo, J. Kukundakwe, M. Naluguza, K. Ssegawa, F. Nsibuka, P. Tuhirirwe, M. Fortunate, J. Acen, J. Achidri, A. Amone, M. Chamai, J. Ditai, M. Kemigisa, M. Kiconco, C. Matama, D. Mbanza, F. Nambaziira, M. Owor Odoi, A. Rweyora, G. Tumwebaze, H. Kalanzi, J. Katabaazi, A. Kiyingi, M. Mbidde, M. Mugenyi, R. Mwebaze, P. Okong, I. Senoga, M. Abwola, D. Baliruno, J. Bwomezi, A. Kasede, M. Mudoola, R. Namisi, F. Ssennono, S. Tuhirwe, G. Abongomera, G. Amone, J. Abach, I. Aciro, B. Arach, P. Kidega, J. Omongin, E. Ocung, W. Odong, A. Philliam, H. Alima, B. Ahimbisibwe, E. Atuhaire, F. Atukunda, G. Bekusike, A. Bulegyeya, D. Kahatano, S. Kamukama, J. Kyoshabire, A. Nassali, A. Mbonye, T. M. Naturinda, A. Nshabohurira, H. Ntawiha, A. Rogers, M. Tibyasa, S. Kiirya, D. Atwongyeire, A. Nankya, C. Draleku, D. Nakiboneka, D. Odoch, L. Lakidi, R. Ruganda, R. Abiriga, M. Mulindwa, F. Balmoi, S. Kafuma, E. Moriku, J. Hakim, A. Reid, E. Chidziva, G. Musoro, C. Warambwa, G. Tinago, S. Mutsai, M. Phiri, S. Mudzingwa, T. Bafana, V. Masore, C. Moyo, R. Nhema, S. Chitongo, Robert Heyderman, Lucky Kabanga, Symon Kaunda, Aubrey Kudzala, Linly Lifa, Jane Mallewa, Mike Moore, Chrissie Mtali, George Musowa, Grace Mwimaniwa, Rosemary Sikwese, Joep van Oosterhout, Milton Ziwoya, H. Chimbaka, B. Chitete, S. Kamanga, T. Kayinga E. Makwakwa, R. Mbiya, M. Mlenga, T. Mphande, C. Mtika, G. Mushani, O. Ndhlovu, M. Ngonga, I. Nkhana, R. Nyirenda, P. Cheruiyot, C. Kwobah, W. Lokitala Ekiru, M. Mokaya, A. Mudogo, A. Nzioka, A. Siika, M. Tanui, S. Wachira, K. Wools-Kaloustian, P. Alipalli, E. Chikatula, J. Kipaila, I. Kunda, S. Lakhi, J. Malama, W. Mufwambi, L. Mulenga, P. Mwaba, E. Mwamba, A. Mweemba, M. Namfukwe, E. Kerukadho, B. Ngwatu, J. Birungi, N. Paton, J. Boles, A. Burke, L. Castle, S. Ghuman, L. Kendall, A. Hoppe, S. Tebbs, M. Thomason, J. Thompson, S. Walker, J. Whittle, H. Wilkes, N. Young, C. Kapuya, F. Kyomuhendo, D. Kyakundi, N. Mkandawire, S. Mulambo, S. Senyonjo, B. Angus, A. Arenas-Pinto, A. Palfreeman, F. Post, D. Ishola, J. Arribas, R. Colebunders, M. Floridia, M. Giuliano, P. Mallon, P. Walsh, M. De Rosa, E. Rinaldi, I. Weller, C. Gilks, J. Hakim, A. Kangewende, S. Lakhi, E. Luyirika, F. Miiro, P. Mwamba, P. Mugyenyi, S. Ojoo, N. Paton, S. Phiri, J. van Oosterhout, A. Siika, S. Walker, A. Wapakabulo, T. Peto, N. French, J. Matenga, G. Cloherty, J. van Wyk, M. Norton, S. Lehrman, P. Lamba, K. Malik, J. Rooney, W. Snowden, J. Villacian

**Affiliations:** 1 Singapore Eye Research Institute, Singapore National Eye Center, Singapore, Singapore; 2 Medical Research Council Clinical Trials Unit, University College London, London, UK; 3 Department of Infectious Disease Epidemiology, London School of Hygiene and Tropical Medicine, London, UK; 4 Department of Infectious Disease, Yong Loo Lin School of Medicine, National University of Singapore, Singapore, Singapore; 5 Program in Health Services & System Research and Center for Quantitative Medicine, Duke-NUS Medical School, Level 6, Academia, Singapore, Singapore; 6 Center for Child Health Research, University of Tampere and Tampere University Hospital, Tampere, Finland

**Keywords:** EQ-5D, Medical outcomes study HIV health survey, Health utility, Mapping

## Abstract

**Background:**

Mapping of health-related quality-of-life measures to health utility values can facilitate cost-utility evaluation. Regression-based methods tend to lead to shrinkage of variance. This study aims to map the Medical Outcomes Study HIV Health Survey (MOS-HIV) to EuroQoL 5 Dimensions (EQ-5D-3 L) utility index, and to characterize the performance of three mapping methods, including ordinary least squares (OLS), equi-percentile method (EPM), and a recently proposed method called Mean Rank Method (MRM).

**Methods:**

This is a secondary analysis of data from a randomized HIV treatment trial. Baseline data from 421 participants were used to develop mapping functions. Follow-up data from 236 participants was used to validate the mapping functions.

**Results:**

In the training dataset, MRM and OLS, but not EPM, reproduced the observed mean utility (0.731). MRM, OLS and EPM under-estimated the standard deviation by 0.3, 26.6 and 1.7%, respectively. MRM had the lowest mean absolute error (0.143) and highest intraclass correlation coefficient (0.723) with the observed utility values, whereas OLS had the lowest mean squared error (0.038) and highest R-squared (0.542). Regressing the MRM- and OLS-mapped utility values upon body mass index and log-viral load gave covariate associations comparable to those estimated from the observed utility data (all *P* > 0.10). EPM did not achieve this property. Findings from the validation data were similar.

**Conclusions:**

Functions are available for mapping the MOS-HIV to the EQ-5D-3 L utility values. MRM and OLS were comparable in terms of agreement with the observed utility values at the individual level. MRM had better performance at the group level in terms of describing the utility distribution.

**Trial registration:**

NCT00988039. Registered 30 September 2009.

**Electronic supplementary material:**

The online version of this article (10.1186/s12955-019-1135-8) contains supplementary material, which is available to authorized users.

## Background

Cost-utility analysis is an important part of the rational development of health care policy and evaluation of medical interventions. It quantifies the cost required for a gain in quality-adjusted life years (QALY) [1] The quality adjustment factor in the estimation of QALY may be obtained from preference-based measures of patient outcomes, such as the EuroQoL 5 Dimensions Questionnaire (EQ-5D) [2] and Health Utilities Index Mark III (HUI3) [3]. Health state valuation studies have provided algorithms to convert the responses to these measures to health utility values, where 1 indicates full health, 0 indicates a state that is not better than death, and negative values indicate health states worse than death [4]. Combining the utility values and patients’ survival duration result in estimates of QALY, which is needed for cost-utility analysis. Availability of utility information is a prerequisite for QALY and cost-utility analyses, but this information is not always available.

Clinical studies often employed quality of life measures that are descriptive, in the sense that they indicate better or worse quality of life but they do not provide a utility value that has a quantitative interpretation for adjusting survival duration to QALY. These descriptive measures are often conceptually overlapping with preference-based measures and empirically correlated with the utility values. In this context, there has been strong interest in developing functions to map descriptive measures to utility values using data from prior studies that included both types of measures [5, 6]. Mapping functions capitalize on descriptive quality of life data and make cost-utility analysis possible when utility data is otherwise not available. Mapping is accepted by the National Institute of Health and Care Excellence Technology Appraisal [7].

Quality of life is an important issue in the care of people living with human immunodeficiency virus (HIV). The EuroQoL Group’s 5 Dimensions 3-level instrument (EQ-5D-3 L) is a commonly used preference-based measure [8]. Its validity and reliability have been demonstrated in various conditions, including in HIV [9]. The responses can be converted to health utility values [4]. The Medical Outcomes Study HIV Health Survey (MOS-HIV) [10, 11] is a descriptive quality of life measures. Both the MOS-HIV and EQ-5D covered multiple health dimensions. The MOS-HIV includes 10 dimensions: general health perceptions, physical functioning, role functioning, pain, social functioning, mental health, energy, health distress, cognitive functioning, and quality of life [11]. The EQ-5D covers five dimensions: mobility, self-care, usual activities, pain/discomfort and anxiety/depression [4, 8]. Despite the more limited scope of the latter, the two measures have sufficient overlap for a conceptual basis for mapping one to the other. A previous study mapped the MOS-HIV to the EQ-5D-3 L utility index [12]. However, the study was oriented towards methodological comparison about the handling of ceiling effects; the functions presented used only one or two significant digits, which is a serious limitation for utility mapping. Availability of an accurate mapping function can make cost-utility analysis in HIV studies become possible even when only MOS-HIV is available.

The ordinary least squares (OLS) is the most commonly used method for mapping a descriptive health measure to a health utility measure [13, 14]. Alternative regression-based methods have been proposed, but there has been no consistent evidence that they performed better than OLS [7, 12, 15–18]. It is well known that OLS mapping under-estimates variability and therefore inflates type 1 errors [5]. Furthermore, OLS mapping tends to under-estimate the health utility of people in good health states and over-estimate it among people in bad health states, which leads to under-estimates of the incremental cost-utility ratio [15, 19–21].

Mapping by the equi-percentile method (EPM) has been successful and popular in education research [5, 22]. It does not suffer from the aforementioned problems. There has been a strong interest in the use of EPM to improve mapping in the health care context [5, 22]. However, EPM is usable only if the cumulative distribution functions (CDF) of the source and target measures are both continuously increasing. Quality of life and health utility measures are often discrete in their distributions, giving CDFs that are step functions. In this situation, Kernel smoothing is required before EPM can be applied [5]. Smoothing health utility and patient reported outcome data is not a simple task. In particular, these measures often have a substantial ceiling effect, which is known to create extra difficulties for smoothing [19–21, 23].

A new mapping method called the Mean Rank Method (MRM) has been recently proposed [21]. Its core idea is similar to EPM and thus it should have similar strengths. However, it does not require smoothing and therefore is much simpler to use than the EPM. One study has mapped the World Health Organization Quality of Life – Brief to the EQ-5D-5 L [21] and another study has mapped the Functional Assessment of Cancer Therapy – Breast (FACT-B) to the EQ-5D-5 L [20] using MRM. Furthermore, the Alzheimer’s Disease Cooperative Study-Activities of Daily Living Inventory (ADCS-ADL) has also been mapped to the Health Utility Index Mark III by the MRM [19]. All three studies demonstrated good performance properties of the MRM at the group level. But the MRM did not out-perform the OLS at the individual level in the FACT-B and ADCS-ADL studies. Further empirical evaluation of the properties of MRM will help to improve understanding of its potentials.

This study therefore aims to map the MOS-HIV to the EQ-5D-3 L utility index, using MRM, OLS and EPM, and to examine the performance of the three mapping functions.

## Methods

### Study participants and study design

From April 2010 to April 2011, HIV-positive adults and adolescents over 12 years old from 14 centers in five sub-Saharan African countries were recruited to the Europe–Africa Research Network for Evaluation of Second-Line Therapy (EARNEST) trial. Details of the trial have been published previously [24]. At weeks 0 (baseline), 48, 96 and 144, the participants completed the MOS-HIV and EQ-5D-3 L and had their body mass index (BMI) measured; HIV viral load was also assayed in real-time at baseline and retrospectively on stored plasma post-baseline. The MOS-HIV and EQ-5D-3 L were in English or in the local languages. To ensure data consistency, only those who filled in the questionnaire in English were included in the present analysis. This secondary analysis was approved by the National University of Singapore Institutional Review Board. The trial protocol was approved by ethics committees in all participating countries and by the research ethics committee of the University College London, UK. All adult participants or the caregivers of participants below 18 years of age provided written informed consent. Patients below 18 years of age also gave assent. Participants were randomized to three different drug regimens. This analysis pooled participants from all trial arms and did not consider the randomization.

### Development and validation datasets

We used data from 421 participants who completed both MOS-HIV and EQ-5D in English at baseline to develop the mapping functions. Among these 421 participants, 236 completed both MOS-HIV and EQ-5D-3 L at one or more of the three scheduled follow-up visits at week 48, 96 or 144. For each of the 236 participants, we randomly selected data from one of the follow-up visits to form a validation dataset, such that it did not involve multiple measurements per person and hence within cluster correlation.

### Questionnaire

The MOS-HIV consists of 35 items and covers 10 dimensions of subjective outcomes [11]. The 10 scores were converted to z-scores and combined to form a Physical Health Summary (PHS) score and a Mental Health Summary (MHS) score that have mean 50 and standard deviation (SD) 10 [25].

The EQ-5D-3 L measures five aspects of health. There are three response options for each dimension (no problem, some problems and extreme problems). The five responses were converted to a utility value using the valuation algorithm of Dolan [4].

### Mapping methods

#### Ordinary least squares mapping (OLS)

Multivariable fractional polynomials (FP) were used to assess the possibility of non-linear relationships between EQ-5D-3 L utility and PHS and MHS and derive the mapping function [26]. The deviance difference was used to guide model selection.

#### Equi-percentile mapping (EPM)

EPM only allows one predictor variable. We explored the relationship between EQ-5D-3 L utility and PHS and MHS using linear regression and FP. A linear regression model showed that one unit increase in PHS and MHS was associated with 0.0083 and 0.0117 unit increase in EQ-5D-3 L utility, respectively. This model explained 52.9% of the variability in the utility values. However, the difference of the two regression coefficients was not statistically significant (*P* = 0.158). Linear regression with a simple mean of PHS and MHS as an independent variable, which we called MOS-score in this article, explained 52.6% of the variability in EQ-5D-3 L utility. Given the similarity in explanatory power, we use the MOS-score to map the MOS-HIV to EQ-5D-3 L. MOS-score was rounded to the nearest integer. This rounding enables the generation of a look-up table for users to apply the mapping result (see Online Additional file 1). This practical purpose was achieved at the expense of generating ties. In contrast, the OLS mapping result appeared as a prediction equation that all predicator values, integer or not, can be plugged into. This issue is addressed in the discussion.

The core concept of the EPM is that the values *x* and *y* are considered equivalent if *F*(*x*) = *P*(*X* ≤ *x*) = *P*(*Y* ≤ *y*) = *G*(*y*), where *F*(*x*) and *G*(*y*) are the cumulative distribution functions (CDF) of the source variable *X* and target variable *Y*, respectively [5, 21, 22]. However, the EPM has no solution if the CDFs are step functions, which is expected in EQ-5D and many quality of life measurement scales such as WHOQOL-BREF or FACT-B [20, 21]. Hence, kernel smoothing is needed [5, 21, 23]. We used the Epanechnikov kernel function to smooth the CDFs [23]. Furthermore, we used the pseudo-data method to mitigate the boundary effect for EQ-5D [27]. After obtaining the smoothed CDFs, we applied the EPM.

#### Mean rank method (MRM)

Conceptually, the MRM is similar to the EPM. Instead of equating the percentiles like EPM, it attempts to equate the ranks. The MRM mapping procedure is as follows [21]:

(1) Let *X* be the predictor variable (MOS-score), whose values are sorted and ranked from the smallest to largest. For tied values, mean of ranks is assigned.

(2) Let *Y* be the target variable (EQ-5D-3 L). Its values are sorted and ranked from the smallest to the largest. Among a set of tied values the ranking is arbitrary.

(3) Each unique *x* value is mapped to the *y* value that has the same rank.

(4) For *n*_*k*_ tied *x* values at the k-th level of unique values in *X* (k = 1,2,…), *x* is mapped to the mean of the *n*_*k*_ consecutive *y* values whose mean of ranks equals the mean ranks of the tied *x* values.

Mathematically, the mean MRM-mapped utility must agree with the mean observed utility [21]. Furthermore, due to the ranking procedure, although there is no direct modelling of association between *X* and *Y*, $\mathrm{rho}\left({\widehat{\mathrm{y}}}_{\mathrm{MRM}},y\right)=\mathrm{rho}\left(x,y\right)$, where ${\widehat{\mathrm{y}}}_{\mathrm{MRM}}$ is the MRM-mapped value and rho is the Spearman rank correlation coefficient, except that ties in *Y* can cause some deviation from this relation [21]. This characteristic is similar to the OLS feature of $\mathrm{r}\left({\widehat{\mathrm{y}}}_{\mathrm{OLS}},y\right)=\mathrm{r}\left(x,y\right)$, where ${\widehat{\mathrm{y}}}_{\mathrm{OLS}}$ is the OLS-mapped value and r is the Pearson’s correlation coefficient. As with EPM, we rounded the MOS-score to the nearest integer and mapped it to the EQ-5D-3 L utility by MRM.

### Evaluation of mapping functions

Different evaluation criteria may have tendency to favour different mapping methods. For example, mean squared errors tends to favour OLS, as minimization of the mean squared errors is the procedure to obtain the OLS estimates. We used multiple evaluation criteria and attempted to interpret the overall profile.

Firstly, from the viewpoint of describing a population, we assessed the mean, SD, and various percentiles of the mapped utilities and checked whether they closely approximated that of the observed utility distribution.

Secondly, we calculated measures of individual-level prediction errors or agreement as compared to the observed EQ-5D-3 L utility values, including mean squared error (MSE), mean absolute error (MAE), intraclass correlation coefficient (ICC), and R2 in correlating mapped utility values to the observed. We used the ANOVA estimator of ICC [6].

Thirdly, estimating utility differences between groups or association with clinical covariates plays a role in cost-utility analysis [21]. We estimated the linear gradients of the mapped utilities in relation to either BMI or log(10)-transformed viral load measured at the time of completing the MOS-HIV and EQ-5D-5 L and compared the parameter estimates with that obtained from the observed EQ-5D-3 L utility data. In order to make the intercepts interpretable, BMI was centered at its 10th percentile and log-viral load was centered at its 90th percentile. As such, the intercepts can be interpreted as the estimated mean utility of people living with HIV in poor health as indicated by low BMI or high viral load. We used Seemingly Unrelated Regression to test the hypotheses of equal regression parameters between each of the mapped utility and the observed utility [28].

## Results

### Participant profile

The left-hand-side panel of Table 1 shows the baseline demographic and clinical characteristics of the 421 participants in the training dataset. The mean age was 37 years; approximately half (48%) of the participants were male. Most participants were from Uganda and Zimbabwe. The mean PHS, MHS, MOS-score and EQ-5D-3 L utility were 46, 48, 47 and 0.731. The MOS-score ranged from 18 to 65; the EQ-5D-3 L utility ranged from − 0.239 to 1. The mapping functions developed were limited to these ranges. The Pearson’s correlation coefficient between MOS-score and EQ-5D-3 L utility was 0.725.

**Table 1:** Descriptive summary of demographic and clinical information and EQ-5D-3 L and MOS-HIV scores in the training (*n* = 421) and validation (*n* = 236) datasets

Variables		Training dataset		Validation dataset	
		Mean	SD	Mean	SD
EQ-5D-3 L		0.731	0.290	0.936	0.162
PHS^a^		46	13	56	9
MHS^a^		48	10	57	7
MOS-score^a^		47	11	57	7
Age		37	11	37	12
CD4 count		101	96	102	96
BMI		21.1	4.4	21.2	4.6
Viral Load (log)		4.8	0.7	4.8	0.6
		N	%	N	%
Country	Uganda	182	43.2	136	57.6
	Zimbabwe	177	42.0	45	19.1
	Zambia	36	8.6	31	13.1
	Kenya	26	6.2	24	10.2
Gender	Male	203	48.2	132	55.9
	Female	218	51.8	104	44.1

^a^*PHS* Physical Health Summary, *MHS* Mental Health Summary, *MOS*-score: mean of PHS and MHS

The right-hand-side panel of Table 1 shows 236 observations that formed the validation dataset. The mean age was 37 years and 56% of the participants were male. The mean PHS, MHS, MOS-score and EQ-5D-3 L utility were 56, 57, 57 and 0.936, respectively. The MOS-score ranged from 23 to 65; the EQ-5D-3 L utility ranged from − 0.163 to 1.

### Development of mapping functions and assessment in the training dataset

#### OLS mapping

The OLS model selected according to deviance difference was:

EQ-5D-3 L = − 0.1278036 + 0.0082448 × PHS + 0.0000373 × MHS^3^ – (8.43/10^6^) × MHS^3^ × ln(MHS).

The model R^2^ was 54.2%.

#### MRM and EPM mapping

The MRM and EPM mapping functions that convert the MOS-score to EQ-5D-3 L utility are provided in Online Supplementary Material 1 as an electronic spreadsheet.

#### Comparison of three mapping functions

**Fig. 1:**
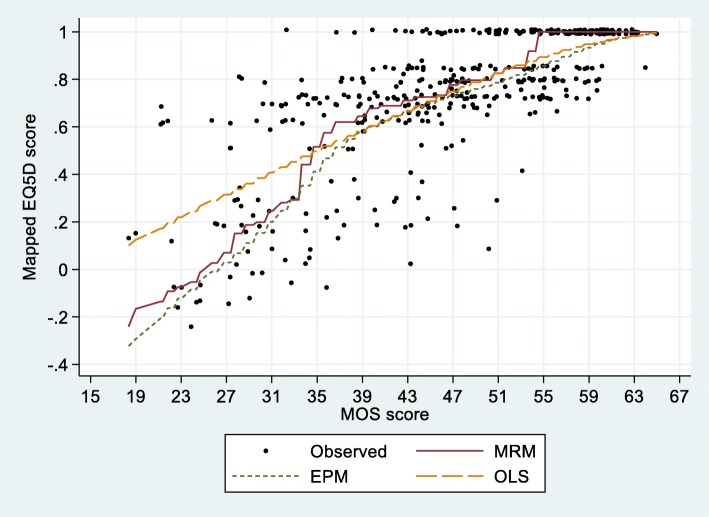
Observed EQ-5D-3 L utility values and mean mapped values obtained from the mean rank method (MRM), equi-percentile method (EPM) and ordinary least square (OLS) in the 18 to 65 range of MOS-score. (With random jittering to avoid over-lapping of data points)

Figure 1 shows the mean mapped values and the observed utility values. For MOS-scores below about 35, the OLS-mapped utility was higher than the MRM-mapped utility, and vice versa. The EPM-mapped values tended to be lower than the OLS- or MRM-mapped values.

The left-hand-side panel of Table 2 shows the details of the distribution of the observed and mapped EQ-5D-3 L utilities. The means of both OLS and MRM agreed closely with the observed value while EPM under-estimated the mean. The SDs of MRM and EPM were similar to the SD of the observed utility values, while OLS under-estimated the SD by 27%. The 75th percentile of the observed EQ-5D-3 L utility reached the full health utility of 1, so did MRM. The maximum of the OLS- and EPM-mapped values were 0.996 and 0.992, respectively. The MRM gave 5th, 10th and 25th percentiles similar to the observed, with absolute errors smaller than 0.02. OLS over-estimated all three percentiles, with errors ranging from 0.058 (25th percentile) to 0.234 (5th percentile). The EPM gave absolute errors ranging from 0.028 (10th percentile) and 0.080 (25th percentile).

**Table 2:** Distribution of observed and mapped EQ-5D utility values in the training (*N* = 421) and validation (*N* = 236) datasets

Statistics^a^	Training dataset				Validation dataset			
	Observed	MRM	OLS	EPM	Observed	MRM	OLS	EPM
Mean	0.731	0.731	0.731	0.668	0.936	0.932	0.896	0.874
SD	0.290	0.289	0.213	0.285	0.162	0.149	0.121	0.153
Minimum	−0.239	−0.239	0.088	−0.322	− 0.163	− 0.074	0.226	− 0.123
P5	0.088	0.070	0.322	0.029	0.516	0.678	0.622	0.601
P10	0.228	0.246	0.401	0.200	0.796	0.726	0.713	0.699
P25	0.656	0.645	0.598	0.576	1.000	0.918	0.888	0.837
Median	0.796	0.796	0.771	0.745	1.000	1.000	0.946	0.933
P75	1.000	1.000	0.917	0.876	1.000	1.000	0.964	0.962
P90	1.000	1.000	0.959	0.95	1.000	1.000	0.971	0.983
P95	1.000	1.000	0.969	0.962	1.000	1.000	0.978	0.989
Maximum	1.000	1.000	0.996	0.992	1.000	1.000	0.997	0.992

^a^*P* Percentile

The left-hand-side panel of Table 3 shows the measures of (dis)agreement between the mapped and observed utilities at the individual level. MRM gave smaller mean absolute error and higher intraclass correlation coefficient than the other two methods did. OLS gave smaller mean squared error and higher R2 than the other two methods did.

**Table 3:** Mean squared errors (MSE), mean absolute errors (MAE), intraclass correlation coefficient (ICC) and R-squared (R2) of the Mean Rank-, OLS- and Equipercentile-mapped EQ-5D-3 L utilities as compared to the observed EQ-5D-3 L utilities in the training (*N* = 421) and validation (*N* = 236) datasets

Method	Training dataset				Validation dataset			
	MSE	MAE	ICC	R2	MSE	MAE	ICC	R2
MRM	0.046	0.143	0.723	0.522	0.019	0.068	0.600	0.361
OLS	0.038	0.147	0.703	0.542	0.019	0.094	0.544	0.356
EPM	0.049	0.158	0.707	0.528	0.023	0.106	0.553	0.376

The left-hand-side panel of Table 4 shows the associations between utilities and BMI and viral load from the regression models. The observed EQ-5D-3 L utility in relation to baseline BMI had an intercept of 0.681 and a slope of 0.009. MRM- and OLS-mapped EQ-5D-3 L utilities showed similar patterns as compared to the observed EQ-5D-3 L’s (model *P* = 0.690 and 0.881, respectively). In contrast, the EPM under-estimated the intercept although the estimated slope was similar to that of the observed EQ-5D-3 L’s (model *P* < 0.001). Analysis of observed and mapped-utility in relation to log-viral load gave similar findings that MRM and OLS produced association pattern comparable to that of the observed EQ-5D-3 L, but EPM did not (*P* < 0.001).

**Table 4:** Relation between observed and mapped EQ-5D-3 L utilities and body mass index and log-transformed viral load in training (*n* = 421) and validation (*n* = 236) datasets

Clinical	Methods	Training dataset					Validation dataset				
feature		Intercept	*P*	Slope	*P*	Model P	Intercept	*P*	Slope	*P*	Model P
BMI	Observed	0.681	Ref.	0.009	Ref.	Ref.	0.913	Ref.	0.003	Ref.	Ref.
	MRM	0.669	0.491	0.011	0.390	0.690	0.909	0.800	0.003	0.959	0.950
	OLS	0.686	0.720	0.008	0.615	0.881	0.879	0.014	0.002	0.724	< 0.001
	EPM	0.610	< 0.001	0.011	0.525	< 0.001	0.853	< 0.001	0.003	0.995	< 0.001
Viral load	Observed	0.683	Ref.	−0.024	Ref.	Ref.	0.935	Ref.	0.002	Ref.	Ref.
	MRM	0.675	0.625	−0.028	0.534	0.824	0.921	0.507	−0.004	0.491	0.783
	OLS	0.687	0.796	−0.022	0.742	0.947	0.891	0.027	−0.002	0.631	0.001
	EPM	0.613	< 0.001	−0.028	0.537	< 0.001	0.866	0.001	−0.002	0.589	< 0.001

### Validation

The right-hand-side panels of Tables 2, 3 and 4 show the results in the analysis of the validation dataset. They mostly agreed with the patterns seen in the training dataset. A difference between the training and validation was that the OLS did not agree with the observed utility pattern in relation to BMI and log-viral load in the validation dataset (right-hand-side of Table 4), despite its agreement in the training dataset. In particular, it under-estimated the intercept in relation to both BMI and log-viral load (each *P* < 0.05 for the intercept; each *P* ≤ 0.001 for the model).

## Discussion

We employed the OLS, EPM and the recently proposed MRM to map the MOS-HIV quality of life scores to EQ-5D-3 L utilities. The OLS is the most commonly used method in the health care context so far [13, 14], but it suffers shrinkage of variance and inaccurate estimation in relation to covariates [5, 15, 21]. There are other regression-based methods, such as the Tobit regression and indirect mapping by multinomial logistic regression [7, 18]. These regression methods do not consistently perform better than the OLS [7, 17, 19].

The applicability of the EPM in the health care context has received a lot of attention. But actual implementation of it has been limited in health research. Two reviews in this field recorded no mapping study that used EPM [13, 14]. This may be related to the nature of CDFs of health utility and descriptive health measures often being step functions, sometimes with a sizeable mass at the ceiling, which makes EPM difficult to implement. The MRM overcomes this complexity by equating mean ranks to handle tied values, instead of smoothing.

One relative strength of OLS is that, unlike the MRM and EPM, it can use multiple predictor variables. In the present study, we used both the PHS and MHS instead of an overall summary score as the OLS predictors. In contrast, we used the mean of PHS and MHS to generate a single predictor variable as the input for MRM and EPM. In this regard, the accuracy of the mapping functions derived may be affected by two factors. Firstly, the association between the observed utilities and PHS and MHS should be approximately equal. As shown earlier, we assessed the equality and found this condition plausible. Secondly, a large deviation of the PHS/MHS ratio from unity (one) may exacerbate the impact of the aforementioned difference, if any. As shown in Table 1, the mean PHS and MHS scores were similar in this study. The mean PHS/MHS ratio was 0.97 in the training dataset. The number of participants who had PHS/MHS ratio < 0.7 or > 1.3 were 40 and 24, respectively. With such small sub-group sample sizes, we refrained from further analyses by sub-groups. While the MRM-derived MOS-HIV to EQ-5D utility mapping function performed well in this study, its performance in other populations will need further assessment in relation to the two conditions aforementioned. Another potential relative strength of the OLS is that the application of an OLS mapping formula does not require rounding of the predictor scores. Unlike many other patient reported outcomes like WHOQOL-BREF or FACT-B which generate integer values, the weighted average procedure in MOS-HIV generates non-integers. For easy utilization of the MRM and EPM mapping results, we rounded the predictor values to integers so that the results can be presented as a simple look-up table. Nevertheless, in this study the OLS did not perform better than the MRM. A previous simulation study has shown that the MRM had mean absolute errors smaller than or equal to OLS even if predictor scores were coarsened to only 10 levels [21]. As such, we expected the rounding to integers to have minimal impact on the accuracy of MRM. Our findings on EPM in this study refer to EPM as applied with MOS-HIV scores rounded to integers.

We have reservations about including demographic and clinical variables in mapping, a practice that has been seen in the health and quality of life literature. This practice changes the research purpose from “mapping a descriptive health measure to a utility measure” to “mapping multiple measures to a utility measure”. The implication of the practice is that the mapping function is not usable unless all the demographic and clinical variables involved in the mapping algorithm are also available.

In the present study, MRM generated a utility distribution that closely reflected the features of the observed utility distribution, including the mean, SD, various percentiles, and the level of ceiling effect. The OLS accurately reproduced the observed mean utility values in the training dataset but under-estimated the mean in the validation dataset. Neither OLS nor EPM were accurate in describing the variability and percentiles at the lower and upper ends of the utility distribution.

As expected, in the training dataset the mean squared error was lowest in the OLS-based mapping. However, in the training dataset both the OLS and EPM had higher mean absolute errors and lower ICC than the MRM. Furthermore, in the validation dataset, the MRM had the same mean squared error as the OLS and better performance according to all other indicators. There was no strong and consistent pattern to indicate whether OLS or MRM was more accurate in making individual-level predictions, but EPM was consistently inferior.

MRM agreed with the observed data in reproducing observed association patterns with clinical covariates. OLS agreed with the observed data in the training dataset but not in the validation dataset. The EPM performed worst in this regard. This suggests that OLS and EPM mapping are less suitable for studies that wish to explore associations. Our study supports the use of MRM, but further validation of this is required.

The mapped values all showed a reasonable degree of accuracy in terms of R2 over 0.5 in the training dataset. This is comparable to a review of mapping studies which showed the R2 in the training datasets in the mapping of disease-specific health measures to utility indices was typically less than 0.5, while mapping of generic health measures typically had within training dataset R2 in the range of 0.4 to 0.6 [13].

We acknowledge that this study has several limitations. Firstly, the MOS-score in the trial only covered the 18 to 65 range. Based on the MOS-HIV reference data [25], the mean and SD are 50 and 10, respectively. The present study covers the lower range quite well (to about − 3 SD) but not the upper range (to about 1.5 SD). This may limit the applicability of the mapping in populations with good health and quality of life. Secondly, the study included only people living with HIV in four African countries. The applicability of the mapping functions in other populations need further evaluation. This includes further evaluation of the relative strength of the association between EQ-5D utilities and PHS and MHS in other populations and the mapping functions’ performance in populations that have PHS/MHS ratio substantially different from unity. Thirdly, the study used the 3-level EQ-5D instead of the latest 5-level EQ-5D (EQ-5D-5 L). Currently there is no official valuation set for mapping the responses to the EQ-5D-5 L to a utility index. Until this is developed, the mapped or observed EQ-5D-3 L utilities will remain useful. In the longer term, updating of the mapping using the EQ-5D-5 L will be needed. Fourthly, our validation dataset was not a randomly selected sample independent of the training dataset, this could have increased the similarity of the training and validation results. However, this impact was minor in this data, because the correlation between EQ-5D-3 L utility at baseline and subsequent visits was weak, ranging from 0.18 (with week 144) to 0.25 (with week 48).

## Conclusions

The MRM performed well in most regards. This method can contribute to future mapping studies. A table for the conversion of MOS-HIV scores to EQ-5D-3 L utility index is provided. This can facilitate cost-utility analysis in the care of people living with HIV.

## Additional file

Additional file 1: MOS-HIV to EQ-5D-3L mapping functions. (XLS 27 kb)

## Abbreviations

ADCS-ADL: Alzheimer’s Disease Cooperative Study-Activities of Daily Living Inventory
BMI: Body mass index
CDF: Cumulative distribution functions
EARNEST: Europe–Africa Research Network for Evaluation of Second-Line Therapy
EPM: Equi-percentile method
EQ-5D: EuroQoL 5 Dimensions
FACT-B: Functional Assessment of Cancer Therapy – Breast
FP: Fractional polynomials
HIV: Human immunodeficiency virus
HUI3: Health Utilities Index Mark III
MHS: Mental Health Summary
MOS-HIV: Medical Outcomes Study HIV Health Survey
MRM: Mean Rank Method
OLS: Ordinary least squares
PHS: Physical Health Summary
QALY: Quality-adjusted life years
SD: Standard deviation
